# Lack of a Functioning P2X7 Receptor Leads to Increased Susceptibility to Toxoplasmic Ileitis

**DOI:** 10.1371/journal.pone.0129048

**Published:** 2015-06-08

**Authors:** Catherine M. Miller, Alana M. Zakrzewski, Dionne P. Robinson, Stephen J. Fuller, Robert A. Walker, Rowan J. Ikin, Shisan J. Bao, Michael E. Grigg, James S. Wiley, Nicholas C. Smith

**Affiliations:** 1 College of Public Health, Medical and Veterinary Sciences, James Cook University, McGregor Rd, Smithfield, Queensland, 4878, Australia; 2 Institute for the Biotechnology of Infectious Diseases, University of Technology, Sydney, PO Box 123, Broadway, New South Wales, 2007, Australia; 3 Molecular Parasitology Section, Laboratory of Parasitic Diseases, National Institute of Allergy and Infectious Diseases, National Institutes of Health, Bethesda, Maryland, 20892–0425, United States of America; 4 Sydney Medical School, Nepean Campus, Nepean Hospital, The University of Sydney, Camperdown, New South Wales, 2006, Australia; 5 Queensland Tropical Health Alliance Research Laboratory, Australian Institute of Tropical Health and Medicine, James Cook University, McGregor Rd, Smithfield, Queensland, 4878, Australia; 6 Discipline of Pathology, School of Medical Science and Bosch Institute, The University of Sydney, Camperdown, New South Wales 2006, Australia; 7 The Florey Institute of Neuroscience and Mental Health, University of Melbourne, Parkville, Victoria, 3010, Australia; University Paris Sud, FRANCE

## Abstract

**Background:**

Oral infection of C57BL/6J mice with the protozoan parasite *Toxoplasma gondii* leads to a lethal inflammatory ileitis.

**Principal Findings:**

Mice lacking the purinergic receptor P2X7R are acutely susceptible to toxoplasmic ileitis, losing significantly more weight than C57BL/6J mice and exhibiting much greater intestinal inflammatory pathology in response to infection with only 10 cysts of *T*. *gondii*. This susceptibility is not dependent on the ability of P2X7R-deficient mice to control the parasite, which they accomplish just as efficiently as C57BL/6J mice. Rather, susceptibility is associated with elevated ileal concentrations of pro-inflammatory cytokines, reactive nitrogen intermediates and altered regulation of elements of NFκB activation in P2X7R-deficient mice.

**Conclusions:**

Our data support the thesis that P2X7R, a well-documented activator of pro-inflammatory cytokine production, also plays an important role in the regulation of intestinal inflammation.

## Introduction

*Toxoplasma gondii* is an intracellular protozoan parasite that enters the body through the mouth, invades the intestinal epithelium, crosses into the lamina propria and then disseminates throughout the body. Thus, like many infectious agents, its first point of contact with the immune system is the intestine, where it provokes a potent pro-inflammatory response that is designed to control parasite replication but can result in immunopathology if not properly regulated [1]. Adequate regulation is the result of interactions between *T*. *gondii*, immune cells, Th1, Th2 and Th17 cytokines (reviewed in [1, 2]).

In C57BL/6 mice, oral infection with 100 tissue cysts of *T*. *gondii* causes a lethal inflammatory ileitis. This is the result of a classic inflammatory cytokine “storm”. Thus, toxoplasmic ileitis, like other inflammatory intestinal diseases, causes necrosis of the villi and mucosa that can be alleviated by depletion of pro-inflammatory cytokines or their receptors; *ie*, IFN-γ, TNF, IL-12, IL-15, IL-17, IL-18 and IL-23 [3–7]. Deletion of the gene for inducible nitric oxide synthase [8] or depletion of nitric oxide [5] also inhibits inflammatory ileitis. With regard to anti-inflammatory cytokines, toxoplasmic ileitis is associated only circumstantially with defective TGF-β signalling [9, 10] but IL-10 plays a key role in ameliorating ileitis; a normally non-pathogenic dose (20 cysts) of *T*. *gondii* can kill mice lacking IL-10 [11].

There are several reasons to believe that the P2X7 receptor (P2X7R) might play an important role in the regulation of intestinal inflammation in response to *T*. *gondii*: first, it is an ancient receptor with a well-documented role in innate immunological control of intracellular infections [12] including *T*. *gondii* [13]; second, it is expressed on a variety of intestinal cells during inflammation [14–17]; and third, it can affect IL-10 production and activation of inducible nitric oxide synthase [18, 19] including in response to *T*. *gondii* [20].

ATP is an important extracellular signal for the immune system, particularly during an inflammatory response. P2X7R, like other purinergic receptors, senses extracellular ATP. However, P2X7R is distinctive from other receptor family members due to its high expression on immune cells and, moreover, its expression is up-regulated by pro-inflammatory cytokines [12]. Not surprisingly, therefore, P2X7R has been implicated in the killing of important intracellular pathogens including *Mycobacteria*, *Chlamydia* and *Leishmania* (reviewed in [12]) and it can also mediate killing of *T*. *gondii*, at least *in vitro* [13]. Furthermore, genetic association studies in diverse human populations show a significant association between resistance to toxoplasmosis and inheritance of a polymorphism in *p2rx7* that enhances function [21].

P2X7R is active in several different cells within the intestine [14–17, 22] and there is evidence that it plays both pro-inflammatory as well as regulatory roles during intestinal inflammation. For example, ATP from intestinal bacteria is known to up-regulate the expression of Th17 cytokines and exacerbate colitis [23]. It has also been shown that P2X7R mediates mast cell-dependent intestinal inflammation [17, 24] and inflammation-induced death of enteric neurons *via* an inflammasome-dependent pathway [16]. P2X7R is also well known as an activator of the inflammasome, a complex of cytosolic proteins that regulates caspase-1 activation and the processing of IL-1β and IL-18 from inactive to active forms. Intestinal expression of IL-1β and IL-18 is enhanced in inflammatory bowel disease (IBD) patients and blocking or deleting IL-18 can reduce intestinal damage in mice [6]. However, the *loss*-of-function Arg-307-Glu polymorphism of P2X7R is associated with IBD, albeit the association is borderline significant (p = 0.06) from a relatively small number of cases and unaccompanied by any association between IBD and other loss- or gain-of-function polymorphisms [25]. This may indicate that the P2X7R has some role in the dampening of inflammatory responses in the gut as well as in the promotion of pro-inflammatory responses that help manage infections. This idea is supported by observations that intestinal epithelial P2X7R levels are low during active IBD but high in control people and IBD patients in a quiescent phase [14].

In this study, we investigated the role of P2X7R in the regulation of toxoplasmic ileitis. Mice lacking P2X7R had similar parasite loads to, but lost weight faster than, C57BL/6J mice and exhibited higher degrees of intestinal pathology. P2X7R-deficient mice also displayed an inability to regulate their intestinal inflammatory cytokine response.

## Results

### Mice lacking functional P2X7 receptors are highly susceptible to toxoplasmic ileitis

**Fig 1:**
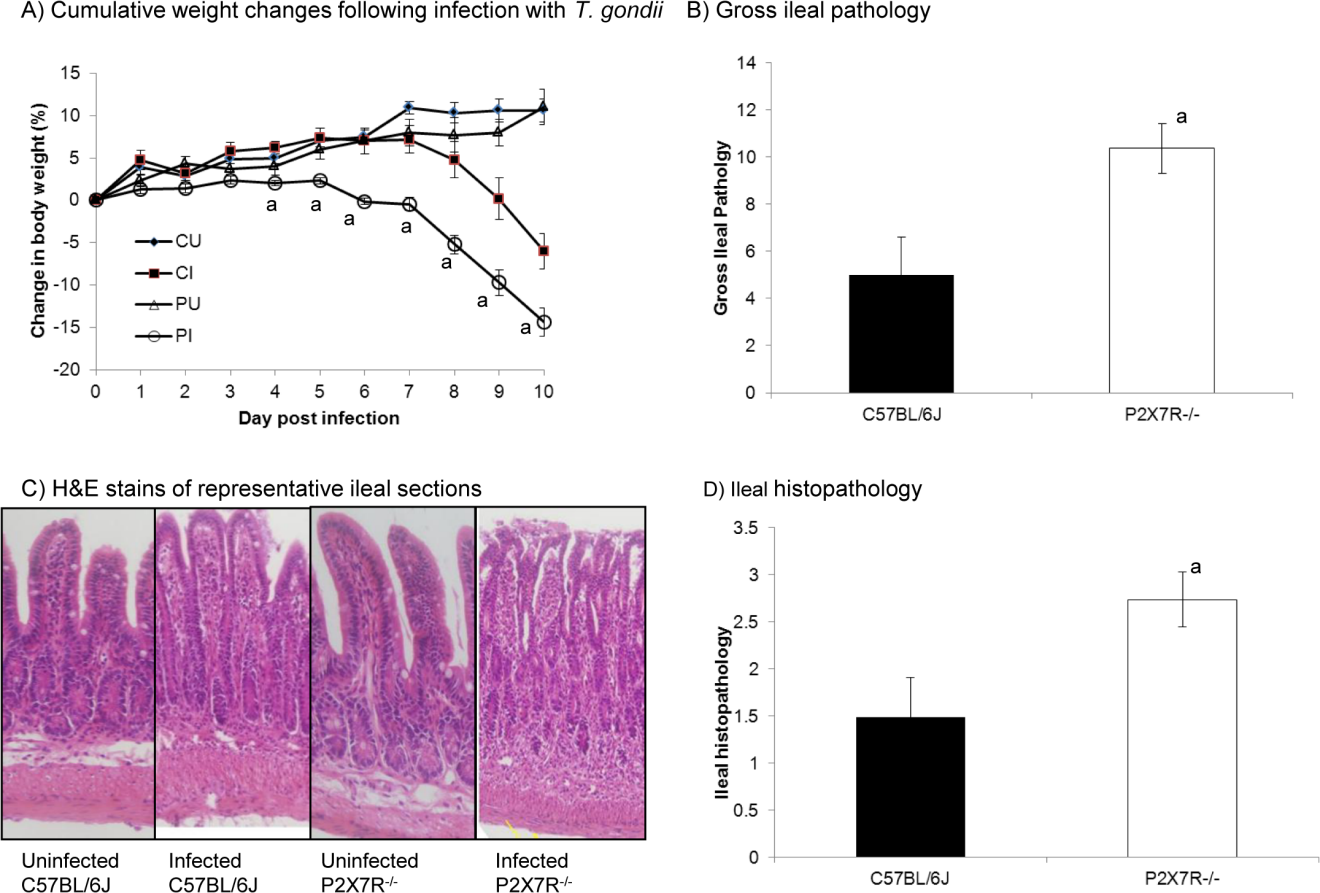
Toxoplasmic ileitis is exacerbated in in P2X7R^-/-^ mice compared with wild type. Male mice (6–8 weeks old) were infected orally with 10 *T*. *gondii* ME49 cysts. (A) Mice were weighed daily for 10 days and cumulative weight changes were calculated and the results presented represent the mean ± SEM of the percentage of weight change relative to starting weight per strain per day from one of six experiments that generated similar data. Infected n = 21/strain; uninfected n = 9/strain. P2X7R^-/-^ mice lost significantly more weight than C57BL/6J mice from day 4 post-infection onward (P<0.005, assessed by multivariate analysis of variance (MANOVA) with days assigned as the within-subjects variable and mouse strain/infection status assigned to the between subjects variable, followed by the assessment of significant interactions within each time point using planned comparisons, i.e., two-way ANOVA coupled to Tukey’s post-hoc test at each day post infection). CU, C57BL/6J uninfected; CI, C57BL/6J infected; PU, P2X7R^-/-^ uninfected; PI, P2X7R^-/-^ infected. (B) Gross ileal pathology was scored on day 8 post-infection based on five observations: consistency of the intestinal contents; absence/presence of blood; absence/presence of pus; degree of swelling; and amount of angiogenesis. This system was adapted from Melgar et al. [60] and was based on an ascending scale of severity, for each parameter, as follows: 0 (no abnormality); 1 (minimal); 2 (moderate); or 3 (severe). The score for each parameter was added to give a total out of a maximum possible score of 15. Gross ileal pathology was also assessed in uninfected mice but no pathology was observed in either strain. Results presented are from one of four experiments that generated similar data. (C, D) Ileal histopathology was evaluated based on five parameters: epithelial cell damage; goblet cell loss; crypt dropout; neutrophil and mononuclear cell infiltration in the submucosa and neutrophil and mononuclear cell infiltration in the muscular layers. Three random fields of view at a 40x magnification were graded on an ascending scale of severity: 0 (no abnormality); 0.25 (minimal); 0.5 (mild); 0.75 (moderate); or 1 (severe) giving a total score out of 5 per mouse. Histopathology was also examined in uninfected mice, however, no pathology was observed in any strain. Photomicrographs of histopathology shown (C) are from single mice and are representative of all mice examined in the group. Results are presented as the mean ± SEM (n = 8) for both strains of mice. Results presented are from one of three experiments that generated similar data. ^a^ Indicates where the score is significantly different from the score for C57BL/6J mice (P<0.05, one-way ANOVA coupled to Tukey’s post-hoc test).

Susceptibility of *p2rx7* gene-deleted mice (P2X7R^-^/^-^) on a C57BL/6J background to toxoplasmic ileitis was assessed relative to C57BL/6J mice by infecting mice with 10 *T*. *gondii* ME49 cysts and monitoring the course of infection. All infected mice exhibited clinical signs of infection and lost weight relative to uninfected mice of the same strain (Fig 1A). P2X7R^-/-^mice lost significantly more weight than C57BL/6J mice beginning on day 4 p.i. and, by day 10 p.i., P2X7R^-/-^ mice had lost 14 ± 1.66% of their body weight versus 6 ± 2.1% for C57BL/6J mice (P<0.005, assessed by multivariate analysis of variance–MANOVA–with days assigned as the within-subjects variable and mouse strain/infection status assigned to the between subjects variable, followed by the assessment of significant interactions within each time point using planned comparisons, *i*.*e*., two-way ANOVA coupled to Tukey’s post-hoc test at each day post-infection)].

**Fig 2:**
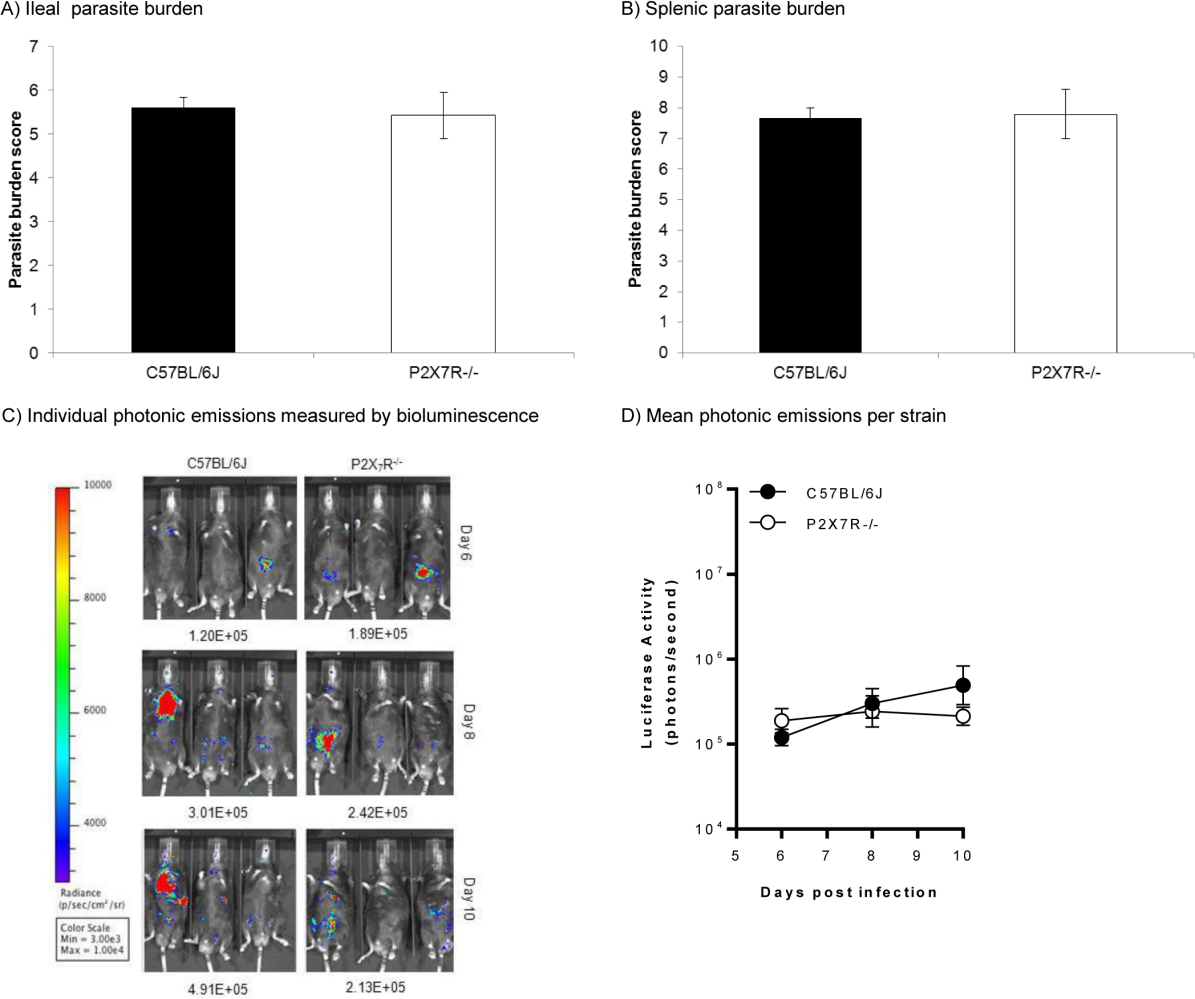
Control of *Toxoplasma gondii* is not altered in P2X7R^-/-^ mice compared with wild type. Male mice (6–8 weeks old) were infected orally with 10 *T*. *gondii* ME49 cysts and euthanased 8 days post-infection. Parasite burden in (A) intestines and (B) spleens of C57BL/6J and P2X7R^-/-^ mice was determined by a microtitre limiting dilution assay as detailed in the Materials and Methods. Results are presented as the mean ± SEM (n = 8) for both strains of mice from one of three experiments that generated similar data. Parasite burden measurements from infected P2X7R^-/-^ mice were not significantly different from infected C57BL/6J mice (one-way ANOVA). Identical results were obtained using the classical plaque-forming assay [61] so these are not shown here. (C, D) In a separate series of experiments, three male and three female mice were infected orally with 5 cysts of luciferase-expressing 76K^GFP-Luc^ *T*. *gondii* and parasite burden was quantified on day 6, 8 and 10 p.i. by firefly luciferase activity using an IVIS BLI system from Caliper Life Sciences as described previously [32]; images of three infected male C57BL/6J and three infected male P2X7R^-/-^ mice are shown (C). Parasite burden measurements (D) from infected P2X7R^-/-^ mice (n = 6) were not significantly different from infected C57BL/6J mice (n = 6) on any of day 6, 8 or 10 p.i. (two-way ANOVA).

Infected P2X7R^-/-^ mice exhibited significantly higher levels of gross ileal pathology with an average score of 10 compared with average scores of 5 for infected C57BL/6J mice (*P*<0.05, one-way ANOVA coupled to Tukey’s post-hoc test) (Fig 1B). P2X7R^-/-^ mice had consistently higher scores in the amounts of swelling, angiogenesis and pus present, as well as consistency of the intestinal contents, which were much more liquefied than the intestinal contents of the C57BL/6J mice. Blood was only rarely observed in severe infections in the P2X7R^-/-^ mice and never in the ileum of infected C57BL/6J mice. No pathology was observed in uninfected mice of any strain. Similarly, a significant increase (*P*<0.05, one-way ANOVA coupled to Tukey’s post-hoc test) in histopathology was observed in infected P2X7R^-/-^ mice compared with infected C57BL/6J mice (Fig 1C and 1D) or uninfected mice of both strains (data not shown). Fig 1C shows representative photomicrograph images of C57BL/6J and P2X7R^-/-^ H&E stained ileal sections from mice that had been infected orally with *T*. *gondii* ME49 for 8 days. A representative photomicrograph portraying a healthy uninfected ileal section is included for reference. By 8 days post infection, both strains of mice showed increased cellular infiltration in the sub-mucosa and the muscle layers compared with uninfected ileal sections of each strain. Furthermore, P2X7R^-/-^ mice lost all discernable villus structure due to severe epithelial cell damage and suffered a complete loss of goblet cells as well as severe crypt drop-out in the lamina propria. This all translated into a much higher histopathology score in infected P2X7R^-/-^ mice (Fig 1D). However, at day 8 p.i., despite the significant increase in intestinal pathology in infected P2X7R^-/-^ mice (*P*<0.05, one-way ANOVA coupled to Tukey’s post-hoc test), there was no significant change in parasite burden in either the intestine or spleen of knockout mice compared with burdens measured in infected C57BL/6J mice (Fig 2A and 2B). Moreover, the dissemination of *T*. *gondii* followed similar kinetics in P2X7R^-/-^ and C57BL/6J mice (Fig 2C and 2D).

### Production of inflammatory cytokines and reactive nitrogen intermediates is elevated in mice lacking functional P2X7 receptors

**Fig 3:**
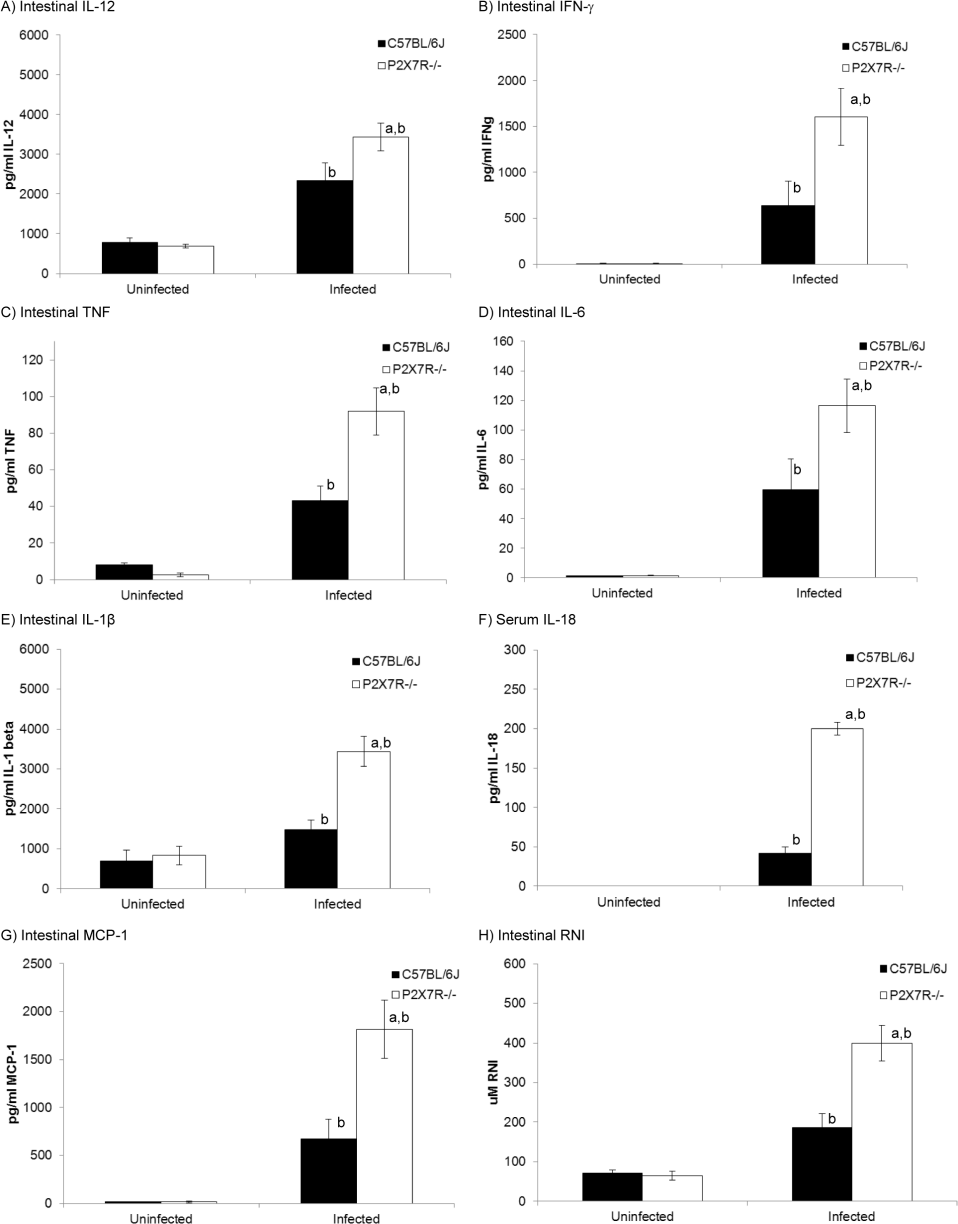
Inflammatory cytokine production is dysregulated in P2X7R^-/-^ mice compared with wild type in association with toxoplasmic ileitis. Male mice (6–8 weeks old) were infected orally with 10 *T*. *gondii* Me49 cysts and euthanased 8 days post-infection. Ileal homogenates were collected for quantification of cytokine levels by ELISA (IL-1β, IL-6, IL-12, TNF, MCP-1, IFN-γ) or Griess assay (reactive nitrogen intermediates) as described in the Materials and Methods. Sera was analyzed for IL-18. Following infection, P2X7R^-/-^ mice (n = 10) exhibited significantly higher levels of (A) IL-12, (B) IFN-γ, (C) TNF, (D) IL-6 (E) IL-1β, (F) IL-18, (G) MCP-1 and (H) reactive nitrogen intermediates compared with infected C57BL/6J (n = 10) or uninfected mice (n = 5) of either strain (P<0.05). Results are presented as the mean ± SEM for one of three experiments that generated similar data. ^a^indicates a significant difference (P<0.05, two-way ANOVA coupled to Tukey’s post-hoc test at each day post infection) compared with infected C57BL/6J mice, ^b^indicates a significant difference (P<0.05, two-way ANOVA coupled to Tukey’s post-hoc test at each day post infection) between infected and uninfected mice of the same strain.

Results in Fig 2 indicated that P2X7R^-/-^ mice were able to control parasite numbers equally as well as C57BL/6J mice, so the more severe pathology observed was not due to lack of control of *T*. *gondii* replication. Production of pro-inflammatory cytokines is essential for control of *T*. *gondii* but, when not regulated, can lead to immunopathology. Therefore, levels of IL-12, IFN-γ, IL-1β, IL-6, TNF and MCP-1 (CCL2), were measured in ileal homogenates from infected P2X7R^-/-^ mice and compared with levels detected in uninfected P2X7R^-/-^ mice, and infected and uninfected C57BL/6J mice. IL-12, IFN-γ and TNF are all crucial in the development of resistance to *T*. *gondii*, however, excessive production of these cytokines has been associated with immunopathology [26–29]. IL-18, IL-1β and IL-6 are also produced in response to *T*. *gondii* as part of an inflammatory response [28, 30, 31] and MCP-1 (CCL2) assists the inflammatory response by recruiting macrophages to sites of inflammation and tissue damage [32]. Ileal homogenates from infected P2X7R^-/-^ mice had significantly elevated levels of IL-12, IFN-γ, TNF, IL-1β, IL-6 and MCP-1 (CCL2) (Fig 3) compared with infected C57BL/6J mice or uninfected mice of either strain (*P*<0.05, two-way ANOVA coupled to Tukey’s post-hoc test). Levels of all these cytokines were similarly elevated in sera of infected P2X7R^-/-^ mice, with the exception of IL-1β, which was not detectable in the sera of any mice studied (data not shown), which is consistent with the findings of others [33]. This may indicate a specific, discrete cellular source for IL-1β, a question that remains unresolved [33]. IL-18 was not detected in the intestinal samples, due to (unresolved) interference of the intestinal samples with the assay. However, IL-18 was detected in sera (Fig 3F) from infected mice and, again, levels of this cytokine were significantly elevated in infected P2X7R^-/-^ mice compared with infected C57BL/6J mice or uninfected mice of either strain (*P*<0.05, two-way ANOVA coupled to Tukey’s post-hoc test). Levels of reactive nitrogen intermediates in ileal homogenates (Fig 3H) and sera (data not shown) were also significantly elevated in infected P2X7R^-/-^ mice compared with infected C57BL/6J mice or uninfected mice of either strain (*P*<0.05, two-way ANOVA coupled to Tukey’s post-hoc test).

Elevated levels of reactive nitrogen intermediates are well known to cause pathology, so we attempted to “rescue” mice from toxoplasmic ileitis by inhibiting and/or quenching reactive nitrogen and oxygen species using methods described previously [5, 8, 34, 35]; however, none of these approaches affected weight loss or intestinal pathology in infected P2X7R^-/-^ mice. All mice started losing weight by day 4 p.i. and lost between 13 and 21% of their bodyweight by day 10 p.i., whether untreated or treated with aminoguanidine, N-monomethyl-L-arginine, or N-acetylcysteine. Production of the anti-inflammatory cytokines, TGF-β and IL-10, was also assessed in ileal homogenates of infected P2X7R^-/-^ mice with levels of these two cytokines varying quite widely from mouse to mouse without being significantly different from background levels seen in uninfected mice of either P2X7R^-/-^ or C57BL/6J mice. Thus, mean and standard error levels (pg/ml) of TGF-β were 200 ± 72 (n = 8) and 212 ± 66 (n = 5) for uninfected C57BL/6J and P2X7R^-/-^ mice, respectively, rising (albeit not statistically significantly) to 400 ± 128 (n = 6) and 436 ± 135 (n = 6) in infected mice of the two respective strains. Mean and standard error levels (pg/ml) of IL-10 were 257 ± 86 (n = 6) and 193 ± 57 (n = 6) for uninfected C57BL/6J and P2X7R^-/-^ mice, respectively, versus 205 ± 90 (n = 6) and 275 ± 115 for infected mice (n = 6).

### Regulation of the NFκB and MAPK pathways is impaired in mice lacking functional P2X7 receptors

**Fig 4:**
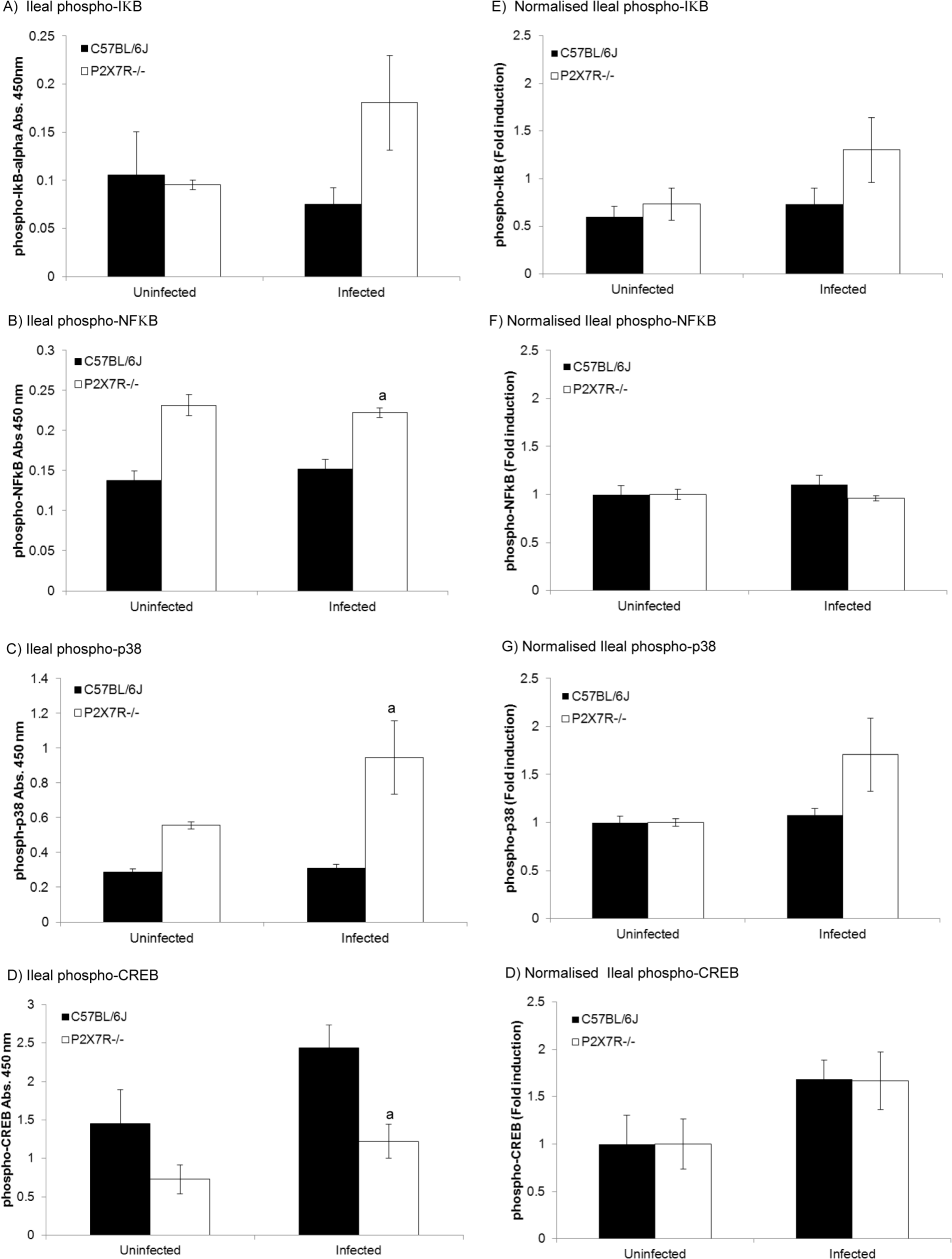
Regulation of transcriptional regulators is impaired in P2X7R^-/-^ mice compared with wild type in association with toxoplasmic ileitis. Male mice (6–8 weeks old) were infected orally with 10 *T*. *gondii* Me49 cysts and euthanased 8 days post-infection. Ileal homogenates were collected for quantification of phospho-IκB, phospho-NFκB, phospho-p38, and phospho-CREB by sandwich ELISA as described in the materials and methods. Following infection, P2X7R^-/-^ mice exhibited significantly higher levels of (A) ileal phospho-IκB, (B) phospho-NFκB, (C) phospho-p38, and significantly lower levels of (D) phospho-CREB compared with infected C57BL/6J or uninfected mice of either strain (^a^ indicates a significant difference, at P<0.05, using a two-way ANOVA coupled to Tukey’s post-hoc test, compared with infected C57BL/6J mice). When the data were normalised against uninfected baseline phospho-protein levels for each mouse strain, no statistically significant differences in phospho-IkB (E) phospho-NFkB (F), phospho-p38 (G) or phospho-CREB (H) were apparent, as measured by fold induction. Results are presented as the mean ± SEM (n = 10 for infected or n = 5 for uninfected mice) for one of three experiments that generated similar data.

Binding of microbial-derived molecules to cell surface receptors on immune cells triggers activation of both NFκB and the mitogen-activated protein kinase (MAPK) signalling pathways. Triggering of the NFκB pathway results in the translocation of NFκB from the cytoplasm to the nucleus where it induces the expression of its target genes [36, 37]. Full transcriptional activity is achieved when phosphorylated NFκB binds CREB binding protein (CBP). The MAPK pathway results in the phosphorylation of p38 MAPK which, in turn, leads to the phosphorylation of CREB *via* MSK1 [38]. Phosphorylated CREB sequesters CBP and inhibits transcription of NFκB-controlled genes thus down-regulating pro-inflammatory cytokine production. To investigate whether the elevated levels of pro-inflammatory cytokines observed in infected P2X7R^-/-^ mice (Fig 3) were the result of differential regulation of these pathways between infected C57BL/6J mice and P2X7R^-/-^ mice, activation of key proteins in the NFκB signalling pathway were tested. Although total baseline levels of CREB were not significantly different between C57BL/6J and P2X7R^-/-^ mice, the baseline levels of NFκB were significantly higher in uninfected P2X7R^-/-^ mice compared with C57BL/6J mice (data not shown). Because phosphorylation at specific residues, rather than overall protein levels, is indicative of activation, phosphorylation of proteins that regulate the inflammatory response, specifically IκB, NFκB, p38 MAPK, and CREB, were measured. No baseline difference was detected between uninfected P2X7R^-/-^ versus C57BL/6J mice for phosphorylated IκB (Fig 4A). However, uninfected P2X7R^-/-^ mice possessed higher baseline levels of phosphorylated NFκB (Fig 4B), phosphorylated p38 MAPK (Fig 4C), and lower levels of the negative regulator, phosphorylated CREB (Fig 4D) than uninfected C57BL/6J, although these results did not reach statistical significance. Following infection, P2X7R^-/-^ mice had statistically increased levels of phosphorylated NFκB (Fig 4B) and p38 MAPK (Fig 4C) as well as reduced levels of phosphorylated CREB when compared against infected C57BL/6J mice (*P*<0.05, two-way ANOVA coupled to Tukey’s post-hoc test). However, when normalized to account for the intrinsic differences observed at baseline between uninfected P2X7R^-/-^ and wild-type C57BL/6J mice, infection did not differentially induce the phosphorylation of NFκB (Fig 4F) or CREB (Fig 4H), but elevated levels of phosphorylated IκB (Fig 4E) and p38 MAPK (Fig 4G) were identified in P2X7R^-/-^ mice, although this did not reach statistical significance using a two-way ANOVA test.

## Discussion

P2X7R^-/-^ mice are acutely susceptible to toxoplasmic ileitis, losing significantly more weight than C57BL/6J (Fig 1) and exhibiting much greater ileal gross pathology and histopathology in response to infection with only 10 cysts of *T*. *gondii* (Fig 1). This susceptibility is not dependent on the ability of P2X7R^-/-^ mice to control the parasite, which they accomplish just as efficiently as C57BL/6J mice. Acute infection with *T*. *gondii* is characterised by the proliferation of rapidly dividing tachyzoites that proliferate inside the cell until the cell membrane ruptures. As each tachyzoite can then go on to invade any nucleated cell, the parasite can disseminate through the body quite quickly and, if not controlled, severe pathology can result. Following oral infection, *T*. *gondii* forms replicative foci within the epithelia and lamina propria of the intestine before disseminating throughout the host’s body [39] albeit sometimes inconsistently [40]. However, we found no significant difference in parasite burden in either the intestine or spleen in infected P2X7R^-/-^ mice compared with infected C57BL/6J mice and the kinetics of dissemination are similar in the two strains (Fig 2). This indicates, first, that P2X7R is not crucial for limiting parasite replication *in vivo* and, second, that the burden of infection with *T*. *gondii per se* is not responsible for the ensuing ileitis.

The unimportance of P2X7R in the control of *T*. *gondii in vivo* is seemingly at odds with demonstrations of P2X7R-dependent killing of *T*. *gondii* tachyzoites *in vitro* [13, 41]. However, it is in keeping with the oft documented observation that parasite burden is rarely associated with toxoplasmic ileitis in a variety of gene knockout mice or mice treated with inhibitors of innate immune effectors [3–11]. Tachyzoites of *T*. *gondii* activate a variety of innate immune effector pathways that affect the viability of the parasite to varying degrees (reviewed in [1]). Two recent studies, for example, documented different emphases on the NLRP1 and NLRP3 inflammasome sensors as important factors in detecting and controlling *T*. *gondii* [30, 33], a point which is particularly pertinent for the overproduction of IL-1β and IL-18, processing of which can be initiated by different inflammasome pathways in response to *T*. *gondii* [30, 33]. Thus, with so many avenues to control *T*. *gondii*, it is not totally surprising that elimination of just one line of attack (in this case P2X7R-dependent killing) may have little effect *in vivo*. Moreover, it is important to remember that a significant feature of toxoplasmic ileitis is the role played by normally commensal bacteria. Oral infection with *T*. *gondii* acts as a trigger for changes in the intestinal bacterial population from >95% Gram-positive to >95% Gram-negative [42]. Bacteria accumulate at sites of mucosal damage and translocate into subepithelial tissues [43] and beyond [44] prolonging the pro-inflammatory response provoked by *T*. *gondii* and leading to immunopathology [45]. It will be intriguing to see if the intestinal flora in P2X7R^-/-^ mice differs from that in C57BL/6J mice in response to the trigger from infection with *T*. *gondii*.

Like *T*. *gondii*, P2X7R has been long recognised as an activator of pro-inflammatory responses [46–49], see [50] for a review). Thus, in the absence of P2X7R, we expected to see reduced production of pro-inflammatory cytokines and reactive nitrogen intermediates, accompanied by diminished intestinal inflammation. However, this was not what we observed. Rather, our results fit with other observations in which reduced P2X7R activity is associated with increased intestinal inflammation and IBD [14]. Moreover, recent work [51] provides a possible explanation for this; whilst normal physiological concentrations of ATP (*ie*, 1-50nm) have little effect on CD4+ T cells or Tregs, and somewhat elevated levels of extracellular ATP (*eg*, 250nm) stimulate the release of pro-inflammatory cytokines, high levels of extracellular ATP (*eg*, 1mM) simultaneously inhibit activated CD4+ T cells and enhance the immunosuppressive activity of Tregs. This phenomenon may be particularly pertinent in toxoplasmic ileitis, which is characterised by a collapse in Treg cell numbers and function [52]. Our data support a role for P2X7R in the regulation of intestinal inflammation since we saw a generalised overproduction of pro-inflammatory cytokines and mediators in P2X7R^-/-^ mice (Fig 3).

Our observations on the intestinal levels of regulators of transcription (Fig 4) provide some clues about how the modulatory effects of P2X7R may be mediated, but do not provide a complete explanation. Extracellular ATP is known to induce activation of CREB *via* the phosphorylation of p38α and MSK-1 [53, 54]. Activated CREB, in turn, binds the transcriptional co-activator, CBP, preventing it from binding with the NFκB subunit, p65, thereby inhibiting expression of pro-inflammatory cytokines [55]. Hence, cells lacking P2X7R may prove less responsive to the sustained build-up of extracellular ATP that could accompany infection with pathogens like *T*. *gondii*. The downstream effect of this would be exactly what we see, that is, decreased levels of phospho-CREB and increased levels of phospho-NFκB following infection; however, NF-κB appears to be constitutively over-produced in P2X7R^-/-^ mice, regardless of infection status. Normalization of the phosphorylation of IκB, NFκB, p38 MAPK, and CREB (Fig 4E–4H) to account for observed baseline differences between P2X7R^-/-^ and C57BL/6J mice revealed that intrinsic differences between the strains correlate with differential phosphorylation of inflammatory regulators, independent of infection. The observed baseline differences in the phosphorylation of NFκB, and CREB (Fig 4B and 4D) between P2X7R^-/-^ and C57BL/6J mice without differential baseline levels of inflammatory mediators (Fig 3; uninfected mice) suggests that these intrinsic differences in the phosphorylation state of NFκB and CREB play a key role following perturbation. Thus, the observed over-production of pro-inflammatory cytokines during toxoplasmic ileitis in P2X7R^-/-^ mice may be partially due to continued activation of the NFκB pathway because not enough CBP is being sequestered by CREB to achieve inhibition of NFκB transcription. Further studies are required to support this hypothesis.

Levels of phosphorylated p38α are also elevated in the intestines of P2X7R^-/-^ mice infected with *T*. *gondii* (Fig 4C). Phosphorylated p38α can stimulate inflammatory and anti-inflammatory pathways through activation of two different downstream kinases [56]. Thus, in an amplification loop, activated p38α activates mitogen-activated protein kinase-activated protein kinase 2 (MK2) by phosphorylation. Activated MK2 then promotes an increase in TNF, IL-1β and IL-6 production through interactions with NFκB [57]. Increased levels of these cytokines then feedback positively on cells and activate the same pathway, amplifying inflammation [56]. In an anti-inflammatory loop, activated p38α phosphorylates mitogen-and stress-activated kinases 1 and 2 (MSK-1/2). MSK-1 and MSK-2 then phosphorylate CREB allowing it to bind CBP and also stimulate the production of dual-specificity protein phosphatase 1 (DUSP 1), which dephosphorylates and inactivates p38 [58]. The elevation in phospho-p38 seen in P2X7R^-/-^ mice suggests impaired regulation of p38 activation in mice without a functional P2X7R, although further work is needed to elucidate the mechanism involved.

In summary, our data support a thesis in which P2X7R, a well-documented activator of pro-inflammatory cytokine production, also plays an important role in the regulation of inflammation in the intestine. Thus, the lack of a functioning P2X7R leads to increased susceptibility to ileitis in mice infected orally with *T*. *gondii* associated with an over-exuberant inflammatory response rather than an inability to control parasite numbers. The increased levels of pro-inflammatory cytokines detected may be the result of impaired regulation of the NFκB and p38 MAPK pathways but whether this implies the existence of a P2X7R-dependent negative feedback mechanism for the inhibition of pro-inflammatory cytokine production or indicates more indirect effects, such as alterations in Treg populations and/or the intestinal microbiome, remains to be demonstrated conclusively.

## Materials and Methods

### Mice and Infections

Pathogen-free, 6–8 week old C57BL/6J mice were obtained from the Animal Resource Centre (Perth, WA, Australia) or from Jackson Laboratories (Bar Harbor, Maine, USA). *p2rx7* gene-deleted mice (P2X7R^-^/^-^) on a C57BL/6J background (back-crossed for at least seven generations]) were originally provided by Pfizer, Inc. (Ann Arbor, MI, USA) and were subsequently bred at the Ernst Facility (University of Technology, Sydney, NSW, Australia) or were purchased from Jackson Laboratories and bred at the Immunogenetics Research Facility (James Cook University, Townsville, Queensland, Australia) or the National Institutes of Health, in Bethesda, USA. Lack of the *p2rx7* gene in the knockout mice was confirmed routinely by PCR using the following primers: Forward 5’-CTATCTCTCCACGACTCACCCCC-3’ and Reverse 5’-TATAATCCCGGGAGGGATACTTGAAGCCACTGTAC-3’ [59].

All animal research was performed in strict accordance and with the approval of: the University of Technology Sydney/Royal North Shore Hospital Animal Care & Ethics Committee (Protocols UTS/ RNSH 0611-042A and UTS ACEC 2008–03); the James Cook University Animal Care & Ethics Committee (Approval Number A1698); and the National Institutes of Health, USA, and Animal Welfare Act (protocol LPD-22E), with mice housed and maintained in an animal facility accredited by the American Association for the Accreditation of Laboratory Animal Care.

Mouse infections were initiated by oral inoculation of cysts of the Type II *T*. *gondii* ME49 or 76K^GFP-Luc^ strains. Cysts were isolated from the brains of chronically infected mice by homogenisation of the whole brain in sterile PBS and centrifugation through a discontinuous Percoll gradient. Suspensions were prepared at the concentrations indicated.

To assess relative susceptibility to *T*. *gondii* infection, male mice of each strain were infected orally with 10 or 20 cysts of *T*. *gondii* ME49, weighed daily and monitored for clinical signs of infection–weight loss, ruffled fur, hunched posture, lethargy and morbidity as per Animal Care & Ethics Protocol stipulations. This experiment was conducted six times; thrice at the University of Technology, Sydney, twice at James Cook University in Cairns, Australia, and once at the National Institutes of Health, USA, in Bethesda, with similar results each time. To assess intestinal pathology and identify aspects of the innate immune response that may be implicated in the increased susceptibility of P2X7R^-/-^ mice to *T*. *gondii* infection, mice were euthanased on day 8 post-infection (p.i.) as per Animal Care & Ethics Protocol stipulations.

### Ileal pathology and histopathology

The ileum was collected from each mouse and scored for gross pathology using a scoring system based on the following five observations: consistency of the intestinal contents; absence/presence of blood; absence/presence of pus; degree of swelling; and amount of angiogenesis. This system was adapted from Melgar *et al*. [60] and was based on an ascending scale of severity, for each parameter, as follows: 0 (no abnormality); 1 (minimal); 2 (moderate); or 3 (severe). The score for each parameter was added to give a total out of a maximum possible score of 15. A 2cm section of the ileum was cut away at the caecum and embedded in paraffin for histology. Sections (5μm) were stained with haematoxylin and eosin (H&E stain) and scored for histopathology by two individuals blinded as to sample identity. Histopathology was assessed using five parameters: epithelial cell damage; goblet cell loss; crypt dropout; neutrophil and mononuclear cell infiltration in the submucosa and neutrophil and mononuclear cell infiltration in the muscular layers. Three random fields of view at a 40x magnification were graded on an ascending scale of severity: 0 (no abnormality); 0.25 (minimal); 0.5 (mild); 0.75 (moderate); or 1 (severe). The grade assigned to each parameter was added to yield a total histopathology score, out of a possible 5, for the tissue within the field of view. An average was determined based on three fields of view from the one tissue. Scores from both investigators were then averaged to reduce any bias or discrepancy in the scoring system. Uninfected mice were included in the experiments as baseline references. The experiment was repeated four times.

### Parasite burden

Ilea and spleens were examined for parasites in tissue sections using immunohistology with a rabbit anti-*T*. *gondii* polyclonal antibody (RayBiotech, Inc., Norcross, GA, USA) as described previously [20]; this preliminary examination confirmed that parasites were infecting similar sites within the intestines and spleens of infected mice in both strains examined (data not shown), often observed in foci in the villi and lamina propria, as recently described [39]. Parasite burden was then measured in the whole spleen and whole intestine of individual mice using a microtitre dilution method adapted from Buffet *et al*. [61], that we have described previously [13]. Briefly, on the day prior to the experiment, 96 well plates were seeded with 5x10^5^ Vero cells/well and allowed to settle overnight. One row was allocated per mouse. Spleens were removed and single-cell suspensions were made by passing through a 70-mm sieve. Cells were pelleted at 1500g, and then resuspended in RPMI 1640 containing 5% FCS at a concentration of 1x10^7^cells/ml. The entire small intestine was removed and processed to ensure consistency between mice strains when measuring parasite burden. Intestines were flushed with Hank’s Buffered Salt Solution (HBSS), chopped into small pieces and incubated in 1640 RPMI/10% Fetal Bovine Serum (FBS) containing 24U Dispase II, 200U collagenase, 100U DNAse at 37°C/5% CO_2_ for 2 hrs. Following incubation the intestines were reduced to single cell suspensions, washed three times by centrifugation then resuspended in 2 ml of fresh 1640 RPMI/10% FBS. Two hundred microliters of spleen or intestinal cell suspension was added to the first well of a 96-well plate and then serially diluted 1/2 across the plate. Plates were incubated at 37°C in 5% CO_2_ for 7 days before wells were examined for the presence of parasites. Relative parasite burden was estimated from the highest dilution in which parasites were visible. An arbitrary score–the “Parasite Burden score”–was allocated based on the last column in which the monolayer was fully lysed. Parasite burden was also determined by using a modified version of the plaque-forming assay described by Pfefferkorn and Pfefferkorn [62]. Five hundred μl aliquots of each intestinal suspension prepared as described above were transferred to flasks containing a confluent monolayer of Vero cells and incubated overnight at 37°C/5% CO_2_. The numbers of plaques formed per flask were then counted and recorded as Plaque Forming Units/500μl (PFU/500μl). These experiments were replicated thrice. In a separate experiment, mice were infected perorally with 5 cysts of luciferase-expressing 76K^GFP-Luc^ *T*. *gondii* and parasite burden was quantified on days 6, 8, and 10 p.i. by firefly luciferase activity using an IVIS BLI system from Caliper Life Sciences as described previously [33].

### Inflammatory mediator analysis

Inflammatory mediator levels were measured in ileal homogenates and in serum in three experiments. An 8 cm section of ileum was removed and flushed with sterile PBS using a syringe fitted with an 18G needle. Once clean, ileal sections were placed in 3 ml of sterile PBS in a 15 ml tube, kept on ice and homogenised using a hand-held glass homogeniser. Samples were then centrifuged at 400g for 10 min. Blood was obtained *via* terminal cardiac puncture, while mice were under anaesthetic, using a 1mL syringe fitted with a 26G needle. The samples were allowed to clot at room temperature before centrifugation at 500*g* for 10 mins. Serum was stored at -20°C until assayed. Ileal supernatants and sera were analysed for cytokine levels using either the BD Cytometric Bead Array Mouse Inflammation Kit, as per the manufacturer’s instructions, or by an Enyzme-Linked Immunosorbent Assay (ELISA).

Levels of IL-6, IL-10, IL-12, monocyte chemoattractant protein-1 (MCP-1 = CCL2) and TNF were measured using a BD Cytometric Bead Array kit (San Diego, CA, USA) according to the manufacturer’s instructions. Briefly, the serum samples and test standards supplied were mixed with cytokine capture beads and phycoerythrin (PE)-conjugated antibodies, and incubated for 2 h in the dark. After washing away unbound sample and reagent, fluorescence was measured in individual standards and samples on a BD LSR-II flow cytometer. Following data acquisition, standard curves were generated and cytokine amounts quantified using FCAP Array, version 1.0.1 (BD, San Diego, CA, USA). Levels of IL-1β, IL-18 and TGF-β were determined using OptEIA kits as per the manufacturer’s instructions (BD, San Diego, CA, USA). Reactive nitrogen intermediate levels were measured in serum and ileal homogenates using the Griess assay as described previously [20].

The endogenous levels of nuclear factor κB (NFκB), phosphorylated NFκB (Ser536), phosphorylated p38 mitogen-activated protein kinase (MAPK (Thr180/Try182)) and phosphorylated inhibitor of κBα (IκB-α (Ser32)) were measured in the intestinal homogenate of infected and uninfected mice using a Cell Signalling Technologies PathScan Inflammation Multi-Target Sandwich ELISA kit as per the manufacturer’s instructions. The endogenous levels of cAMP response elements (CRE) binding protein (CREB) and phosphorylated-CREB (Ser133) were measured in the intestinal homogenate of infected and uninfected mice using Cell Signalling Technologies PathScan Sandwich ELISA kits as per the manufacturer’s instructions.

### Statistical analyses

The statistical significance of differences between groups was determined using a one-way ANOVA coupled to Tukey’s post-hoc test for the pathology scores and the intestinal and splenic parasite burdens. Changes in body mass were assessed by multivariate analysis of variance (MANOVA) with days assigned as the within-subjects variable and mouse strain/infection status assigned to the between subjects variable, followed by the assessment of significant interactions within each time point using planned comparisons (two-way ANOVA at each day post infection). A two-way ANOVA was used to assess luciferase activity in the bioluminescence parasite burden assay with day post infection and mouse strain as the independent variables. The inflammatory mediator and phosphorylated protein measurements were assessed by two-way ANOVA with infection status and mouse strain as the independent variables. In all two-way ANOVA assessments, the post-hoc analyses of pairwise multiple comparisons were performed using Tukey’s post-hoc test. A *P* value of <0.05 was considered significant.
